# JEPETTO: a Cytoscape plugin for gene set enrichment and topological analysis based on interaction networks

**DOI:** 10.1093/bioinformatics/btt732

**Published:** 2013-12-19

**Authors:** Charles Winterhalter, Paweł Widera, Natalio Krasnogor

**Affiliations:** ^1^Institute of Systems & Synthetic Biology, University of Evry Val-d’Essonne, 91000 Evry, France and ^2^School of Computing Science, Newcastle University, Newcastle NE1 7RU, UK

## Abstract

**Summary:** JEPETTO (Java Enrichment of Pathways Extended To TOpology) is a Cytoscape 3.x plugin performing integrative human gene set analysis. It identifies functional associations between genes and known cellular pathways, and processes using protein interaction networks and topological analysis. The plugin integrates information from three separate web servers we published previously, specializing in enrichment analysis, pathways expansion and topological matching. This integration substantially simplifies the analysis of user gene sets and the interpretation of the results. We demonstrate the utility of the JEPETTO plugin on a set of misregulated genes associated with Alzheimer’s disease.

**Availability:** Source code and binaries are freely available for download at http://apps.cytoscape.org/apps/jepetto, implemented in Java and multi-platform. Installable directly via Cytoscape plugin manager. Released under the GNU General Public Licence.

**Contact:** jepetto.plugin@gmail.com

**Supplementary information:** Supplementary data are available at *Bioinformatics* online.

## 1 INTRODUCTION

The integration of heterogeneous data derived from functional genomics experiments is an essential step in providing insights into biological systems behaviour, especially disease-related processes. Nowadays this integration is becoming easier, thanks to extendable network analysis platforms such as Cytoscape (Shannon *et al.*, 2003). Although several existing Cytoscape plugins do functional analysis, not many go beyond performing a ‘term-based’ search or link ontology. In contrast, JEPETTO integrates gene sets with pathways and the molecular interaction networks they are embedded in. It uses information from three different web servers to perform network enrichment, pathways expansion and topological analysis.

## 2 IMPLEMENTATION

### 2.1 Input

Our plugin operates on target gene set provided by a user. The list of gene names can be given directly or imported from an existing network created in Cytoscape (many gene identifier formats are accepted, e.g. *Ensembl*, *HGNC*, *Entrez*, *UniProt*). The other main parameters are the reference annotation database (*KEGG*, *BioCarta*, *GO*, *InterPro*, etc.) and the molecular interaction network (*STRING* or a user-defined network).

### 2.2 Processing

**Fig. 1.**
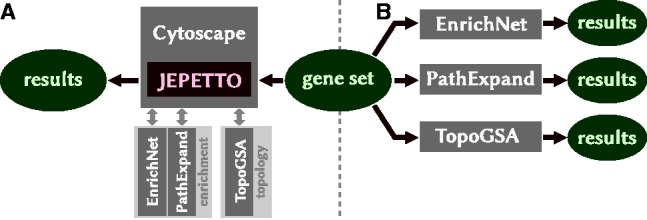
Plugin architecture. (**A**) analysis via JEPETTO (single input/output), (**B**) analysis via existing web servers (multiple input/output points)

There are two types of analysis available in JEPETTO based on: (1) enrichment or (2) topology. For the enrichment analysis, *EnrichNet* and *PathExpand* web servers are used and the topological analysis is performed with *TopoGSA*. The plugin communicates with the web servers as shown in Figure 1A, and integrates the results within the Cytoscape environment. This single-point integration eliminates the need to use the web servers individually and simplifies repeated analysis on previously obtained results.

*EnrichNet* (Glaab *et al.*, 2012) maps the input gene set onto a molecular interaction network and, using a random walk, scores distances between the genes and pathways/processes in a reference database. This network-based association score (XD-score) is relative to the average distance to all pathways and represents a deviation (positive or negative) from the average distance. As an option, EnrichNet provides XD-scores for 60 human tissues, derived from tissue-specific gene expression data.

*PathExpand* (Glaab *et al.*, 2010a) maps the input pathway/process onto the human protein–protein interaction network and extends it with proteins that (1) are strongly associated with the pathway nodes; and (2) increase the pathway compactness by connecting its disconnected members. The exact expansion acceptance criteria can be modified in the plugin advanced options.

*TopoGSA* (Glaab *et al.*, 2010b) maps the input gene set on an interaction network, computes its topological signature and compares it against signatures of pathways/processes in a reference database. The topological signature is built from five distinct properties, among others: network density, centrality of nodes in the network or their tendency to form clusters.

### 2.3 Output

**Fig. 2.**
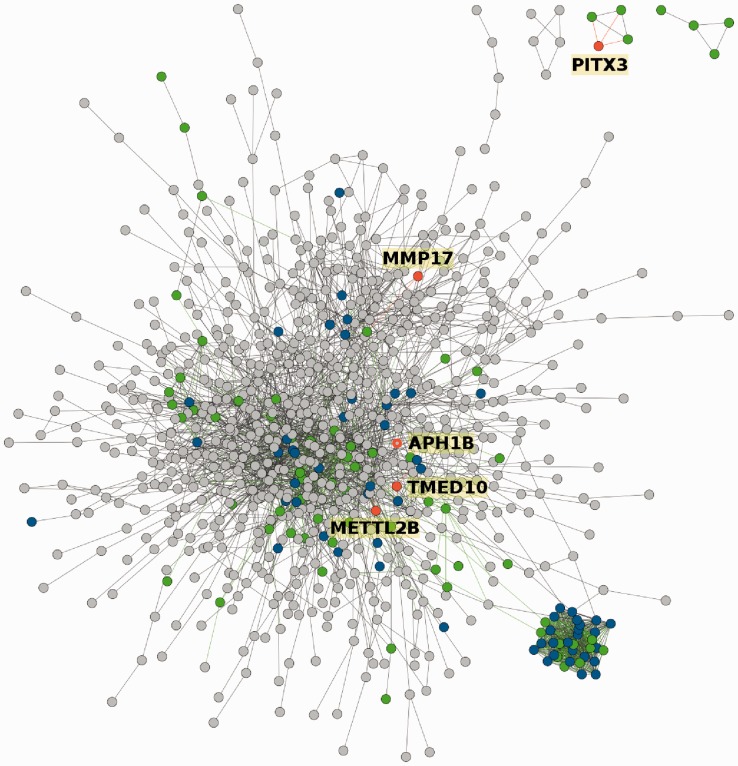
Target gene set within Alzheimer’s disease environmental network. Grey nodes represent the input set, green the pathway and blue the overlap between them. Orange nodes represent the expansion. Interactions between the input set and added nodes are highlighted: edges to pathway nodes are green and edges to expansion nodes are orange

**Fig. 3.**
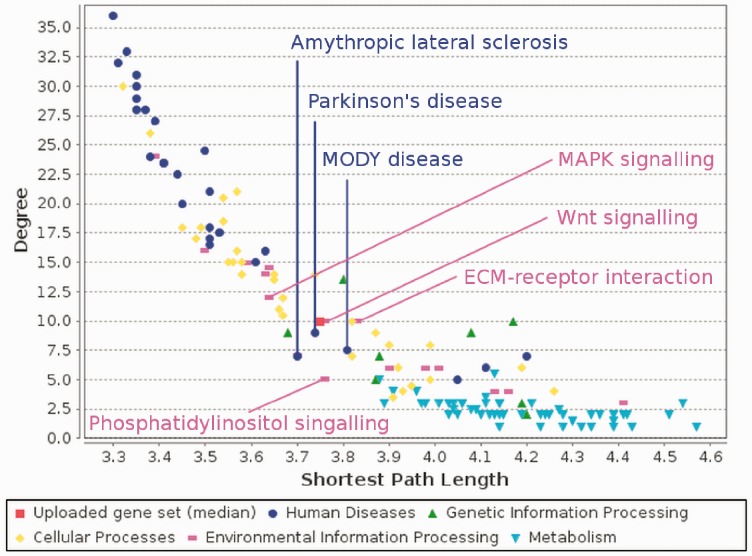
Comparative analysis of topological properties. The red square next to the *Wnt* signalling pathway represents the target network

The enrichment analysis finds pathways significantly associated with the input gene set (in terms of XD-score). When a pathway is selected, its expansion is constructed and a network of interactions between the gene set, pathway and expansion is generated (see Fig. 2). The topology analysis finds pathways with a pattern of interactions most similar to those in the input gene set and visually compares their topological properties (see Fig. 3).

## 3 CASE STUDY

We have retrieved the Alzheimer’s disease-related set of genes from *Phenopedia* (http://www.hugenavigator.net). The enrichment analysis of this set assigned the highest XD-score (1.94) to the Alzheimer’s disease pathway. The pathway was expanded and the disease environmental network (see Fig. 2) was generated. The following genes were added as the expansion: *TMED10*, *METTL2B*, *APH1B*, *MMP17* and *PITX3*.

The first three of these genes have been experimentally characterized as direct Alzheimer’s cofactors (additional details in Supplementary Information). *MMP17* protein family was found to play a role in β-amyloid proteins degradation related to the increase of mitogen-activated protein kinase (MAPK), the main trigger of the Alzheimer’s disease (Yoshiyama *et al.*, 2000). *PITX3* is involved in transcription of a micro-RNA that is known to have reduced expression levels in Parkinson’s diseased brains and may also contribute to the Alzheimer’s pathology development (Shioya *et al.*, 2010).

The topological analysis of the largest connected component of the Alzheimer’s disease environmental network confirmed the previous findings. Among the most topologically similar pathways were *Wnt* signalling pathway related to *MMP17* and Parkinson’s disease pathway related to *PITX3*. It also highlighted similarity to sclerosis and diabetes and several environmental information processing pathways (see Fig. 3 and Supplementary Information).

## 4 SUMMARY

JEPETTO integrates three different network-centric human gene set analysis methods under a single interface of the Cytoscape 3.x environment. It performs enrichment and topological analysis based on the interaction networks. It displays the target gene set within its interaction environment and identifies possible gene cofactors and topologically related pathways and processes that are unlikely to be detected using traditional term-based analysis.

In the case study of the Alzheimer’s disease-associated genes, JEPETTO was able to identify a number of known disease cofactors and suggested directions for further investigation.

*Funding*: Engineering and Physical Sciences Research Council [EP/J004111/1].

*Conflict of Interest*: none declared.

## Supplementary Material

Supplementary Data
