# Supplementary material for: JEPETTO: a Cytoscape plugin for gene set enrichment and topological analysis based on interaction networks
Source: Bioinformatics. 2013 Dec 19;30(7):1029–30. doi: 10.1093/bioinformatics/btt732 (PMC3967109; doi:10.1093/bioinformatics/btt732)
Supplement: Supplementary Data [file supp_30_7_1029__index.html]

JEPETTO: a Cytoscape plugin for gene set enrichment and topological analysis based on interaction networks — JEPETTO: a Cytoscape plugin for gene set enrichment and topological analysis based on interaction networks — JEPETTO: a Cytoscape plugin for gene set enrichment and topological analysis based on interaction networks — Supplementary Data 

# JEPETTO: a Cytoscape plugin for gene set enrichment and topological analysis based on interaction networks

## Supplementary Data

files

**Files in this Data Supplement:**

- Supplementary Data - pdf file
