# Supplementary material for: JEPETTO: a Cytoscape plugin for gene set enrichment and topological analysis based on interaction networks
Source: Bioinformatics. 2013 Dec 19;30(7):1029–30. doi: 10.1093/bioinformatics/btt732 (PMC3967109; doi:10.1093/bioinformatics/btt732)
Supplement: Supplementary Data [file supp_btt732_supplementary_information.pdf]

BIOINFORMATICS

Vol. 00 no. 00 2013
Pages 1–5

Supplementary Information

JEPETTO: a Cytoscape plugin for gene set enrichment and topological analysis based on interaction networks

Charles Winterhalter[2], Paweł Widera[1] and Natalio Krasnogor[1]

[1]School of Computing Science, Newcastle University, Newcastle, NE1 7RU, UK.
[2]University of Evry Val-d'Essonne, 91000, Evry, FR.

Received on XXXXX; revised on XXXXX; accepted on XXXXX

Associate Editor: XXXXXXX

1 ALZHEIMER'S DISEASE CASE STUDY

In this study, we use JEPETTO to perform an integrated analysis with a target set of misregulated genes associated with Alzheimer's disease. Alzheimer is the most common type of dementia resulting in the brain degeneration. The desease is one of the leading causes of death for individuals over the age of 65. Dysregulation of the brain cell functions slowly generates a set of harmful symptoms: memory loss, personality changes, misorientation and others. The disease worsens as it progresses and although some treatments exist, they do not tackle the long-term effects (Thies and Bleiler, 2011). Alzheimer's disease remains incurable. Therefore, it is important to identify new components that may be involved in the disease development or the potential targets for a cure.

2 DATA SOURCES

In the following case study, we used JEPETTO to analyse a set of genes related to Alzheimer's disease. The gene set associated with the disease was retrieved from *Phenopedia* (Yu *et al.*, 2010), part of the Human Genome Epidemiology (HuGE) encyclopedia. It contained 1551 human genes found in 2705 PubMed publications, related with Alzheimer's disease. The complete list of genes in this study is provided in Table 4 (last page).

3 WORKFLOW

JEPETTO offers two types of analysis. The **enrichment analysis** finds pathways strongly associated with a query gene set in the context of an interaction network, while **topology analysis** finds pathways sharing a similar set of topological features.

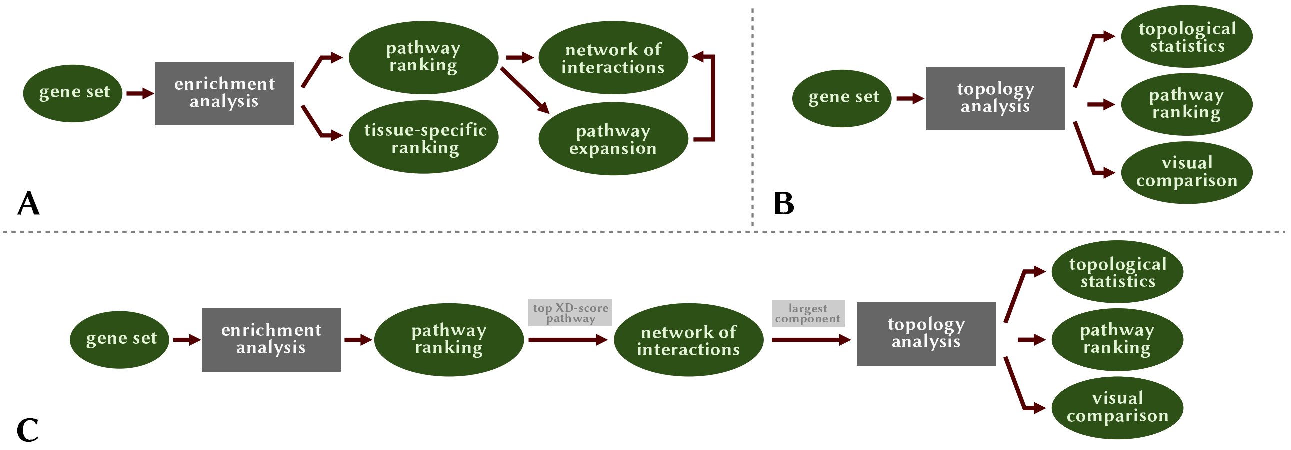

Fig. 1. Examples of JEPETTO's workflow: (**A**) enrichment analysis provides general and tissue-specific ranking of pathways ordered by XD-score and a network of interaction between the input gene set, a selected pathway and the selected pathway expansion (if found), (**B**) topology analysis provides topological signature of the input gene set interactions compared to randomly sampled same-size networks, ranking of pathways ordered by topological similarity to the input gene set and visual comparison of pathway topological properties, (**C**) combined analysis used in the Alzheimer's disease case study; a network of interactions is generated using a pathway with the highest XD-score and the largest connected component of the network is used as input to topology analysis.

© Oxford University Press 2013.

Each type of analysis is performed independently of the other, but depending on the research question at hand, different workflows could be used to combine them.

If a user is interested in the gene set functional interpretation, she will perform the enrichment analysis and focus on pathways with the highest scores. She could generate the network of interaction for each pathway of interest and analyse the overlap with the gene set and the protein-encoding genes added as the path expansion.

Sometimes a different reference database might be preferred. *KEGG* works well for the enzymatic pathways but to describe the gene set in terms of association to the biological processes, *GO* database is more adequate. The same is true for molecular functions or sub-cellular localisation.

If a user wants to check if the pattern of interactions between a set of genes is unusual, she will perform the topology analysis and focus on the topological statistics. In particular, she might compare the topological signature of the gene set interactions against the interactions expected by chance.

The user might be interested in exploration of the regulatory mechanisms and use pathways ranking to indicate new directions of research. Moreover, she could use the visual comparison to examine the distribution of selected topological properties across pathways.

Figure 1 shows the information flow in the enrichment analysis, topology analysis and a combination of the two that we used in this case study. We were interested in capturing the pattern of interactions between the input gene set and the Alzheimer's disease pathway. Therefore, in our workflow, the largest connected component of the generated network of interactions, is used as an input to the topology analysis (see Figure 1C). This is by no means the only possible approach and JEPETTO allows users to follow different workflows.

4 PATHWAYS ENRICHMENT ANALYSIS

In the first step of the enrichment analysis with JEPETTO, the target gene set was mapped onto the *String* interaction network. Out of the 1551 gene identifiers, 1079 were successfully mapped and used in the further analysis. The closest pathways and cellular processes from *KEGG* were identified using **XD-score** (see Table 1).

XD-score uses a random walk in the molecular interaction network to determine a distance between the mapped genes and the pathways in the reference database. The score is relative to the average random walk distance between the mapped genes and all the pathways (background model). Positive values of XD-score imply stronger than average association between genes and the pathway, while negative values imply a below average, weak association.

As it was expected, Alzheimer's disease signalling pathway appeared at the top of the pathways ranking (see Figure 2). It had high XD-score of 1.944, over three times above the significance threshold of 0.61 found by the regression fit (equivalent to adjusted for multiple comparisons Fisher test q-value of 0.05 incremented by the upper bound of the 95% confidence interval to compensate for model parameters uncertainty).

Among other top ranked pathways we found the Parkinson's disease, two cytochrome P450 metabolism pathways, malaria, two types of diabetes, bladder and thyroid cancers, asthma and sclerosis. Several of these pathways had a Fisher test q-value > 0.05 and due to small overlap size, it is unlikely that the functional link to the

pathway	XD-score	q-value	overlap
Alzheimer's disease	1.94363	0.00000	61/138
Parkinson's disease	1.68315	0.00030	34/99
Drug metabolism - cytochrome P450	1.63051	0.00088	10/17
Adipocytokine signaling pathway	1.61004	0.00000	31/57
Oxidative phosphorylation	1.47101	0.01427	27/94
Metabolism of xenobiotics by cytochrome P450	1.43247	0.00088	11/20
Malaria	1.32922	0.00010	20/42
Allograft rejection	1.27581	0.00071	13/25
Huntington's disease	1.23351	0.00080	44/149
Type I diabetes mellitus	1.23075	0.00030	15/29
Asthma	1.15382	0.01046	9/19
Graft-versus-host disease	1.11136	0.00216	12/25
Complement and coagulation cascades	1.08489	0.00002	28/65
Arachidonic acid metabolism	1.05812	0.01046	11/26
Renin-angiotensin system	1.03942	0.04537	7/16
Type II diabetes mellitus	0.91851	0.00036	19/43
Steroid hormone biosynthesis	0.91025	0.09053	6/15
Thyroid cancer	0.88697	0.00024	14/25
Prion diseases	0.85760	0.00030	17/35
NOD-like receptor signaling pathway	0.82540	0.00058	23/59
Linoleic acid metabolism	0.79510	0.08463	5/11
Bladder cancer	0.78203	0.03185	13/38
Amyotrophic lateral sclerosis (ALS)	0.72818	0.00675	17/47
Chagas disease	0.67119	0.00001	38/99
Retinol metabolism	0.65284	0.11294	5/12
Leishmaniasis	0.65217	0.00009	26/62
Notch signaling pathway	0.64745	0.03164	14/42

Table 1. Strongly associated pathways found in KEGG. The XD-score determines the significance of the association. The q-value determines the significance of the overlap (Fisher test). Last column shows number of overlapping genes vs size of the pathway.

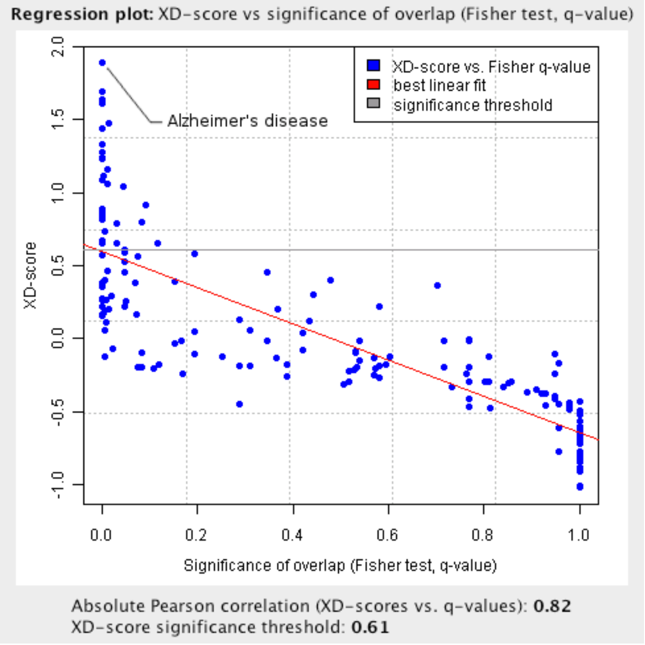

Fig. 2. JEPETTO screenshot of the regression plot between XD-score and the overlap significance (q-value). Each dot in the regression plot represents a pathway or process predicted in the enrichment analysis as related to the Alzheimer's disease target gene set. Best linear fit is shown with a red line. The XD-score significance threshold is shown with a grey horizontal line. The Alzheimer's disease pathway visible at the top had XD-score $= 1.944$ and q-value $< 10^5$.

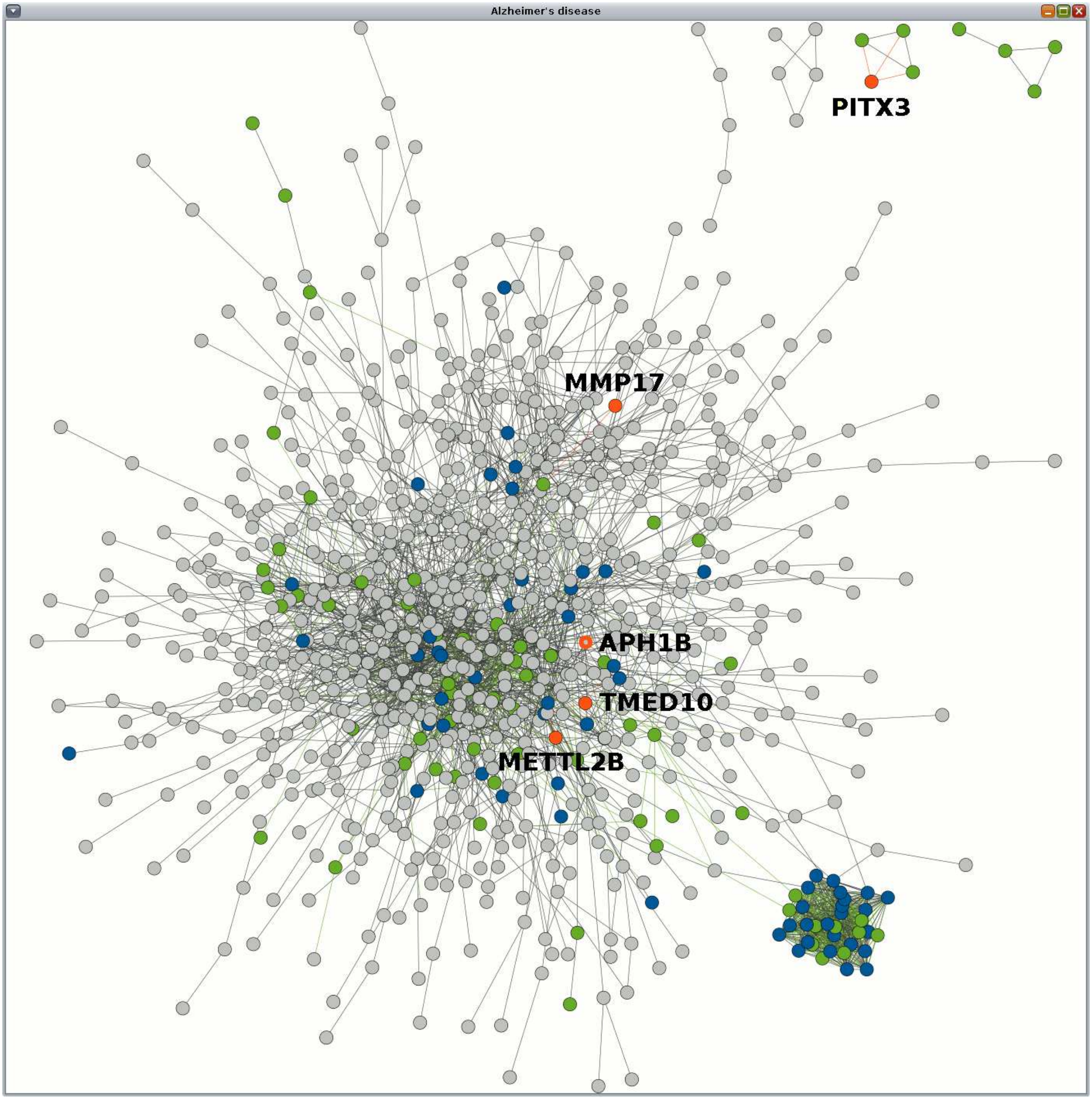

Fig. 3. Target gene set within Alzheimer's disease environmental network. Node colours are: grey for the genes in the target set, blue for the overlap between the pathway and the target gene set, green for the pathway specific components and orange for pathway/process expansion (all expansion nodes are also labelled). The edge colours are: green for interactions between the input set and the pathway, orange for interactions between the input set and the expansion and grey for others. Only connected components larger than 3 nodes are shown to improve clarity.

input gene set would have been found by traditional term overlap analysis methods that ignore the topology.

To further strengthen the results, JEPETTO *expands* the selected pathway with strongly associated proteins. [1] The resulting Alzheimer's disease gene environmental network is shown in Figure 3. There are two main gene clusters visible in the network: (1) mainly green nodes on the right, specific to the associated pathway and (2) a mix of pathway specific genes and genes from the target set on the right. The labelled orange genes in between the two clusters (*APH1B*, *METTL2B*, *MMP17*, *TMED10* and *PTIX3*) are the predicted path expansions.

[1] As this process is based on human protein-protein interaction network, the results for non-human gene sets have to be interpreted with care.

Curiously enough, *APH1B* was already present in the input gene set and was "rediscovered" by the path expansion algorithm from the analysis of the Alzheimer's disease pathway interactions.

APH1B is a gene that may display a rare polymorphism observed in Alzheimer's disease associated populations, increasing the disease susceptibility through interactions with the Apolipoprotein E. The principle is that *APH1B* is an important subunit of the γ-secretase complex which is known to produce amyloid β-peptides (main molecular actor in Alzheimer's disease). *APH1B* single nucleotide polymorphism implies a native interaction with the Apolipoprotein E dysregulating neuronal processes, specifically the ones observed in Alzheimer's disease population (Poli *et al.*, 2008).

METTL2B corresponds to a putative methyltransferase interacting with mutated presenilins. Presenilin genes are recognized as being part of the major components involving an early-onset of Alzheimer's disease. In addition, methyltransferase proteins and preselinins have been found to work together in Alzheimer's genesis (Zhang *et al.*, 2001).

TMED10, also known as *TMP21*, is a negative regulator of the amyloid β-peptide production. During the Alzheimer's disease development amyloid β-peptides are aggregated on a rolling basis and quickly become highly neurotoxic agents (Bromley-Brits and Song, 2012; Cohena *et al.*, 2013). An inhibition of *TMED10* can thus only enhance the molecule aggregation, therefore increase Alzheimer's development and symptomatic effects.

This means that without any prior knowledge of the disease specific mechanisms, JEPETTO was able to automatically identify three genes known to be direct cofactors of the Alzheimer's disease.

The other two genes are not yet known to play any role in the Alzheimer's pathology development. However, the proteins from the matrix metalloproteinase *MMP17* family are capable of degrading the β-amyloid proteins and it is speculated that distribution of MMPs in the brain matter is a part of Alzheimer's pathomechanism (Yoshiyama *et al.*, 2000). Therefore, further studies on *MMP17* as a disease cofactor and its role in β-amyloid proteins accumulation might be interesting.

The last gene in the expansion set, *PITX3*, is an auto-regulated component producing micro-RNAs. It transcribes *miR-133b* which expression level is known to be down-regulated in Parkinson's diseased brains (Shioya *et al.*, 2010). For this reason, it might also contribute to the Alzheimer's pathology development. Particularly in early stages of the disease, the analysis of *PITX3* expression profile may help to understand its genesis.

5 TOPOLOGICAL ANALYSIS

The enrichment analysis was complemented with a network topology analysis. As input, we used the largest connected component (767 nodes) of the enriched network. The topological properties of the network were compared to the properties of random interaction networks of the same size. The results revealed significant differences in the topological signatures (see Table 2).

The average shortest path length is smaller than in random networks which indicates closer interactions. This is confirmed by over two times higher average node degree which additionally reveals more dense interactions between the target genes. Also the average node betweenness centrality is almost three times higher which signals the presence of more central nodes (hubs) in the target network. The difference in eigenvector centrality suggest more variety in node importance, although that difference is not significant in comparison to the background network. Interestingly, genes in the target set are not more likely to form clusters, as the average clustering coefficient is the same as for random networks. These results support the hypothesis that the target network is specific and interactions between its genes are unlike those commonly present in the background network as a whole.

	Topological properties				
**Network**	**SPL**	**BC**	**D**	**CC**	**EC**
**Target**	3.81	45287	18.4	0.11	0.04
**Random**	4.13 ± 0.02	14189 ± 2492	8.14 ± 0.66	0.11 ± 0.01	0.02 ± 0.00
**Background**	4.12 ± 0.94	14669 ± 68893	8.27 ± 16.2	0.11 ± 0.21	0.02 ± 0.04

Table 2. Enriched Alzheimer's disease network topological properties. Topological properties are: shortest path length (SPL), betweenness centrality (BC), node degree (D), clustering coefficient (CC), eigenvector centrality (EC). For random networks mean values from a simulation with 100 samples are reported.

The topological signature of the interactions in the enriched network was then compared to those of known pathways and biological processes. We searched the *KEGG* database for the closest topological matches. Table 3 shows the list of the most similar biological mechanisms found.

The closest topological match was the *Wnt* signalling pathway ($score = 0.05$). It is involved in tissue development but also participates in genesis of various tumours and pathologies (Goodwin and D'Amore, 2002). In particular, it acts jointly with the *β-catenin* signalling pathway in the regulation of development of blood vessels in the central nervous system during angiogenesis (Daneman *et al.*, 2009). Blood vessels differential maturation or regression is known to be partially modulated by *MMP17* (Sounni *et al.*, 2011). Therefore there seems to be a link between *MMP17* and Alzheimer's early onset, as indicated by the pathway expansion.

Name	Score
Wnt signaling pathway	0.05
Ubiquitin mediated proteolysis	0.08
Tight junction	0.09
Melanogenesis	0.10
ECM-receptor interaction	0.10
Parkinson's disease	0.11
Regulation of actin cytoskeleton	0.11
Natural killer cell mediated cytotoxicity	0.12
Amyotrophic lateral sclerosis	0.13
Axon guidance	0.13
Long-term depression	0.14
Gap junction	0.14
Maturity onset diabetes of the young	0.15
GnRH signaling pathway	0.15
MAPK signaling pathway	0.15
TGF-beta signaling pathway	0.17

Table 3. Closest topological matches found in KEGG. The distance score associated with each match is a normalised sum of ranks computed from differences between the topological properties.

Also the previously found link to Parkinson's disease (*PITX3* pathway expansion) is reflected in the topological similarity of the Parkinson's pathway ($score = 0.11$).

Another interesting top match was the maturity onset diabetes of the young (MODY) pathway ($score = 0.15$). A link between the diseases has been confirmed by recent experiments on Alzheimer transgenic and diabetic mice (Takeda *et al.*, 2010), where an increase in the *amyloid-*β aggregation caused by diabetes, resulted in increased Alzheimer's effects.

To complete the topological analysis, we have performed a visual comparison using node degree and shortest path length, two properties with values significantly different from those obtained by chance for the random networks. Figure 4 shows the target network in relation to other *KEGG* pathways and processes. Among the most similar diseases we found, except of the Parkinson's disease and MODY discussed above, the myotrophic lateral sclerosis. Interestingly, sclerosis as a neuro-degenerative process shares several biological mechanisms with Alzheimer's disease, e.g. *SIRT1*, a lastingness effector, protects neurons from degeneration in both diseases (Kim *et al.*, 2007).

The closest environmental information processing pathways in Figure 4 were the previously discussed *Wnt* signalling, together with phosphatidylinositol signalling and ECM-receptor interaction. Phosphatidylinositol compounds take part in many cellular processes and for some of them a metabolism imbalance has been observed during Alzheimer's disease pathogenesis (Landman *et al.*, 2006). The extracellular matrix (ECM) components interacts with amyloid precursor proteins (APP) and aberration in that process is associated with a deposition of β-amyloid senile plaques, a hallmark pathology of the Alzheimer's disease Small *et al.* (1993). Interestingly, MAPK signalling pathway, known to contribute to the brain inflammation in Alzheimer's disease Munoz and Ammit (2010), was found closer to the target network in the comparative analysis than in the pathway ranking.

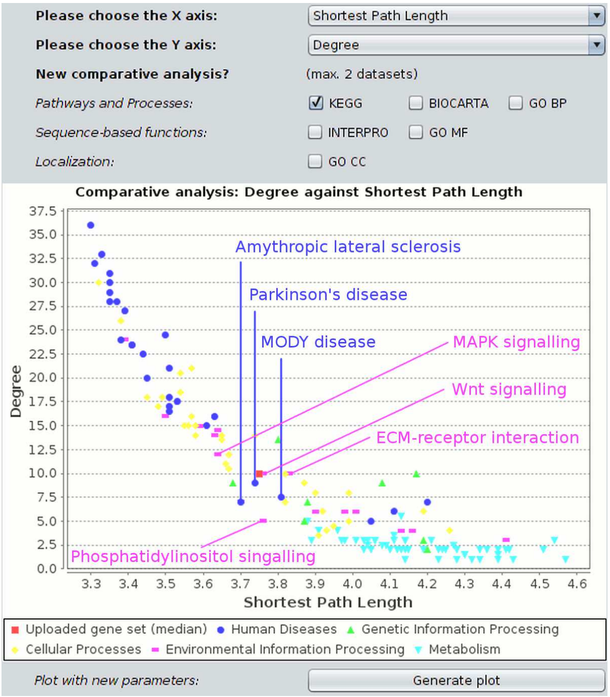

Fig. 4. JEPETTO results panel displaying comparative analysis of topological properties. The target network is represented with a red square.

6 SUMMARY

To conclude, by using JEPETTO on a new Alzheimer's disease associated gene set we were able to identify correctly three known disease cofactors (*APH1B*, *METTL2B*, *TMED10*) and a number of candidate genes and pathways that may be involved in the pathology genesis worth further investigation (e.g. *MMP17* and *Wnt* singalling pathway or *PITX3* and Parkinson's disease pathway).

Funding: This work was supported by the Engineering and Physical Sciences Research Council [EP/J004111/1, EP/H000968/1].

REFERENCES

Bromley-Brits, K. and Song, W. (2012). The role of TMP21 in trafficking and amyloid- precursor protein (APP) processing in Alzheimer's disease. *Curr. Alzheimer Res.*, **4**(9), 411–24.

Cohena, S., *et al.* (2013). Proliferation of amyloid-beta 42 aggregates occurs through a secondary nucleation mechanism. *PNAS*, **24**(110), 9758–9763.

Daneman, R., *et al.* (2009). Wnt/-catenin signaling is required for CNS, but not non-CNS, angiogenesis. *Proc. Natl. Acad. Sci.*, **106**(2), 641–646.

Goodwin, A. and D'Amore, P. (2002). Wnt signaling in the vasculature. *Angiogenesis*, **5**, 1–9.

Kim, D., *et al.* (2007). SIRT1 deacetylase protects against neurodegeneration in models for Alzheimer's disease and amyotrophic lateral sclerosis. *EMBO J.*, **26**(13), 3169–3179.

Landman, N., *et al.* (2006). Presenilin mutations linked to familial Alzheimer's disease cause an imbalance in phosphatidylinositol 4,5-bisphosphate metabolism. *Proc. Natl. Acad. Sci.*, **103**(51), 19524–19529.

Munoz, L. and Ammit, A. J. (2010). Targeting p38 MAPK pathway for the treatment of Alzheimer's disease. *Neuropharmacology*, **58**(3), 561–568.

Poli, M., *et al.* (2008). Interaction between the APOE epsilon4 allele and the APH-1b c + 651T > G SNP in Alzheimer's disease. *Neurobiol. Aging*, **10**(29), 1494–1501.

Shioya, M., *et al.* (2010). Aberrant microRNA expression in the brains of neurodegenerative diseases: miR-29a decreased in Alzheimer disease brains targets neurone navigator 3. *Neuropath. Appl. Neuro.*, **36**(4), 320.

Small, D. H., *et al.* (1993). The Role of Extracellular Matrix in the Processing of the Amyloid Protein Precursor of Alzheimer's Diseasea. *Annals of the New York Academy of Sciences*, **695**(1), 169–174.

Sounni, N. E., *et al.* (2011). MT-MMPs as regulators of vessel stability associated with angiogenesis. *Front. Pharmacol.*, **2**(111).

Takeda, S., *et al.* (2010). Diabetes-accelerated memory dysfunction via cerebrovascular inflammation and A deposition in an Alzheimer mouse model with diabetes. *Proc. Natl. Acad. Sci.*, **107**(15), 7036–7041.

Thies, W. and Bleiler, L. (2011). 2011 Alzheimers disease facts and figures. *Alzheimer's and Dementia*, **2**(7), 208–244.

Yoshiyama, Y., *et al.* (2000). Selective distribution of matrix metalloproteinase-3 (MMP-3) in Alzheimer's disease brain. *Acta Neuropathol.*, **99**(2), 91–95.

Yu, W., *et al.* (2010). Phenopedia and Genopedia: disease-centered and gene-centered views of the evolving knowledge of human genetic associations. *Bioinformatics*, **26**(1), 145–146.

Zhang, S., *et al.* (2001). Identification of a novel family of putative methyltransferases that interact with human and Drosophila presenilins. *Gene*, **1**(280), 135–44.

Names of genes associated with Alzheimer's disease

APOE, MAPT, ACE, BDNF, CLU, PSEN1, PICALM, IL1A, CR1, MTHFR, IL6, IL1B, SORL1, TNF, APP, BCHE, A2M, PRNP, SLC6A4, CYP46A1, CTSD, IDE, CST3, TOMM40, BIN1, PSEN2, LRP1, IL10, BACE1, GRN, ESR1, TF, PON1, HFE, COMT, GAB2, ABCA1, CALHM1, NOS3, LDLR, ABCA7, CHAT, APOC1, NCSTN, SERPINA3, C9orf72, HTR2A, CD33, VEGFA, CYP2D6, MME, TFAM, TGFB1, PLAU, LRRK2, CETP, CD2AP, UBQLN1, SORCS1, CYP19A1, DAPK1, EPHA1, MS4A6A, PTGS2, MPO, WWC1, CH25H, EXOC3L2, VDR, APOA1, IL1RN, ESR2, LPL, MMP3, TREM2, PPARG, ABCB1, PIN1, NEDD9, OLR1, CCL2, PPARA, CHRNB2, HMGCR, APBB1, PCDH11X, GSK3B, CTNNA3, TNK1, GSTO1, GRIN2B, HLA-A, ICAM1, IFNG, ALOX5, STH, GBA, CRP, TFCP2, GOLM1, PON2, LMNA, FAS, IL8, AR, LIPC, MAOA, LRPAP1, MMP9, ATP7B, PCK1, TLR4, RELN, ALDH2, CHRNA7, GSTO2, ADAM10, BACE2, ECE1, APOD, GSTP1, GSTT1, CDC2, NR1H2, FTO, CBS, GSTM1, CFH, HMOX1, SIRT1, PRND, DNMBP, MTHFD1L, CCR5, AGER, DRD3, DRD4, TCN1, SERPINE1, NOS1, MTR, ACHE, KIF11, KLC1, IL18, LIPA, ND1, PARK2, NPC1, OGG1, MS4A4A, SOAT1, TLR2, THRA, CXCL12, BLMH, SNCA, DRD2, CYP26A1, NQO1, CYP17A1, DLST, CHRNA4, NAT2, GAPDH, GAPDHS, TARDBP, MS4A6E, APBB2, HHEX, GSTM3, HSPG2, HTR2C, CHRFAM7A, LRAT, ABCG2, VEGF, ADAM12, CAT, PGBD1, APH1B, GALP, ZNF224, CAMK2G, XRCC1, VLDLR, USF1, UCHL1, PLA2G7, SCARB1, NRXN1, IL33, CD14, MYH13, HTR6, APOB, HLA-DRA, HSD11B1, HSPA1A, APOC3, IGF1R, IL2, IL4, IGF1, IL1R1, ANK3, DISC1, FGF1, SORCS3, FYN, CDK5, SIGMAR1, CDKN2A, PPARGC1A, CHRM2, CALHM3, CREB1, DBH, CTSS, CCR2, PARP1, AGT, ABCA2, SLC6A3, SLC18A3, SST, SELP, MS4A4E, LOC645503, TP53, TPH1, SOD2, SREBF1, HTRA1, PLAT, PPARD, PSENEN, PPP3R1, CFB, ATXN1, SORT1, RXRA, PTPRA, APH1A, NTRK1, NTRK2, CALHM2, NGF, MMP1, KNS2, INS, LTA, ACT, LCAT, ARMS2, SERPINA13, LEPR, LHCGR, MAOB, LRP2, IRS1, KCNMA1, ACAT1, IL6R, MOBP, COX1, COX2, MIF, MBL2, TRNK, NGFR, TRNG, MTHFD1, NOS2A, ND2, ND3, MTRR, ATP6, ATP8, ASCC1, MLXIPL, SLC11A2, OPRL1, NPY, NOTCH4, PLCE1, PLA2G4A, PIK3R1, PLG, PLTP, PON3, APBB1IP, ACTA1, PVRL2, GPAM, ALDH18A1, PTEN, BCR, REN, RBP4, SAA1, SAA2, CCL3, FRMD4A, TAPBPL, PPP2R2B, PRKACB, PRKG1, DNAJC12, C10orf2, SAR1A, SREBF2, TCF7L2, TACR2, TAP2, COX3, ND2, ND1, SUPV3L1, C2, C3, TRAF2, C1R, TP73, TNFRSF1B, TIMP1, MARK4, SELE, SCD, PCDH15, TRAK2, SNAP25, ERBB4, F2, F5, CCNY, DRD1, DNMT3B, AGTR1, DYRK1A, AHR, CSNK1D, ADRA2A, CRYAB, CREM, VTI1A, ADRB3, ADRB1, ADRB2, CYP3A4, CYP2C9, CYP2C18, DHCR24, ABCC2, CNTF, CHUK, APOA5, OPRS1, SPON1, PITRM1, PRDX3, STX6, NR1H3, EBF3, FUS, FRAP1, FSHR, GAD2, TRPC4AP, ABCA12, GHR, AKT1, CARD8, MSRB2, ARL5B, FGFR2, ALOX5AP, SIRT3, SIRT2, DKK1, GPX1, GALNT2, GSTA4, NRG1, HMOX2, APOC4, IGF2, APOC2, LRRTM3, HLA-DRB1, HSPA2, HMGCS2, TNFRSF6B, CAV1, CASP1, NAT1, IL18R1, SGPL1, MS4A1, KL, PDLIM1, EIF2AK3, LIPG, FOXP2, ABCG1, CLOCK, ECE2, DOPEY2, NCAPD2, CACNA1A, LRP8, ATP8B4, BICC1, CUBN, UBE2D1, USF2, TTR, CCR2, YWHAZ, UCP2, UCP3, CASP7, TET1, CAMK2A, RASSF4, COL25A1, CASR, TTBK1, AFAP1L2, MYPN, SLC9A7, HINT2, C10orf11, IMMP2L, PIP4K2B, PLA2G6, PRPF18, RRP1, AKR1CL2, FAM107B, IRS4, PSRC1, C10orf33, FBXO18, LINGO1, ATAD1, PLXDC2, ALG10, MASTL, ATG4C, ARHGAP19, RBM17, NDST2, LIPF, ITGA8, GCM1, SHANK3, LMO4, KNDC1, ITPRIP, TRIM8, NIPA2, KAZALD1, EPC1, SLC25A16, TNKS2, C6orf27, FGF23, ITIH5, HMGA2, CALML3, CLPTM1L, ANP32A, DTNBP1, PPP1R1B, ARID5B, LOXL4, ZDHHC16, DYDC2, PCGF5, GPR123, LZTS2, PHYHIPL, CUL2, MRPL43, NETO1, ZNF239, SLC25A28, ZRSR2, PRDM2, CAST, PCDH11Y, UGT2B7, ZNF33B, MZF1, CA9, VSNL1, VWF, WNT8B, WRN, XBP1, XPNPEP1, XPO1, SFTPA2, TXN, TXNRD1, TYMS, LOC729983, UBA52P1, UBE2A, VCAM1, VCL, VDAC2, UBE2I, C9, CCDC6, NCOA4, CXXC6, C10orf57, HKDC1, C10orf79, STAM, THNSL1, CALCR, SYNPO2L, OBFC1, TRAPPC6A, APOO, ALDH5A1, BAT1, MMEL1, CACNB2, IL1R2, MALL, VKORC1, CUEDC2, CACNA1C, VGF, VIM, C10orf76, RNF219, ADIPOR2, MCPH1, NARS2, C10orf68, HPS6, CCDC134, C10orf119, P2RY14, DCLRE1A, NR1H4, CCS, SLK, PHACTR2, BMS1, SPOCK2, KEAP1, RHOBTB1, NUAK1, CDC20, SPAG6, KIF20B, CD40LG, TRAF4, SEC24C, SH3PXD2A, ARHGAP12, OPN4, FHL5, CHST3, MAPK8IP1, SLC25A27, GDF15, VPS4B, ENTPD1, BAG3, PTGES, VPS26A, MINPP1, NR1D1, SMC3, KCNQ4, ACBD5, NEURL, FAM113B, EXO1, ATPAF2, IL1RL1, IDI2, DDX21, CD4, PTPLA, LGI1, CD5L, NOLC1, PCDH21, CADPS2, NRXN3, ADIPOQ, NRXN2, DLG5, CD9, TCEAL1, TXNL1, ANUBL1, RGN, ANKRD30A, INA, ACTN3, BTRC, CCND3, NRP1, CDK5R1, LDB1, SYNJ1, CDC123, AQP10, BTAF1, PAPSS2, PKD2L1, RUNX2, TNFSF11, RUNX1, PDE8B, TP63, SOCS1, IRS2, EIF3A, BECN1, PEA15, DGAT1, TNFRSF11A, IL18RAP, ADAM9, HRK, GBF1, HTR7, APOA4, KCNK18, WHAMMP3, IRF8, CYP26C1, VWA2, C10orf112, CFI, HNRNPCL1, IFIT2, IFIT1, IFIT3, HSPA4, HSPA5, HSPA6, HSPA8, HSPA9, HSPB2, HSPD1, DNAJB1, HMGCS1, HSPA1B, HSPA1L, HSD17B1, DNAJB2, IFNGR1, OSTN, IGF2R, IGFBP1, IGFBP2, IGFBP3, IGFBP4, IGFBP5, IGFBP6, RGSL1, IL1RAP, IL4R, SHROOM2, IL2RA, FASLG, IL3, HNF4A, HNMT, HNRNPA1, HNRNPF, HNRNPH3, APOF, ACACB, APBA1, HOXD13, HP, HPCAL1, HPGD, APCS, HPS1, APEX1, CFHR1, KCNIP2, A2MP, NXPH1, SH3KBP1, HABP2, HIP1, HK1, ANXA7, ANXA11, HLA-DOA, HLA-DOB, HLA-DPA1, HLA-DPB1, HLA-DQA1, CXCL1, GRPR, GSR, GSS, GSTA1, GSTA2, GSTA3, GSTM4, GSTM5, GSTZ1, BLNK, CARD10, SNX8, POMT2, GPX4, RABGEF1, POLL, SIT1, PDCD4, GCLC, C10orf28, GRB2, GRIA1, CCDC22, SLC25A4, GRIN1, KIN, NT5C2, SCAP, FMO3, CSTF2T, ICOSLG, KCTD2, FOLR3, MYST4, FOSB, TSPAN15, GTPBP4, PRG1, MLYCD, PPIL2, ALOX12, IFIT5, FRK, ALOX15, FGG, COG2, CPEB3, NLRP1, NLGN1, INPP5F, FKBP5, ZNF365, GSTA5, KIF6, TMEM217, BEND7, FDPS, FGA, FGB, SEPHS1, ERCC4, FGF2, FGF13, FGFR1, FGFR3, ZNF292, KIAA0913, WAPAL, FOXO1, PPRC1, FOXO3, GGA3, CYFIP1, RRP12, ARC, ASTN2, GHRH, GHRHR, AP3M1, ABL2, ACAD8, GHITM, SNX5, HTRA2, GLRX, GLUD1, GNB3, ABO, GPR3, C10orf125, JAKMIP3, THEM5, DLL1, GRK5, GC, GCK, GCKR, CNNM1, MYOF, GDF2, GDF10, GDI2, GFAP, PGBD3, DAOA, GFRA1, GGT1, B4GALT1, GH1, GATA1, FBXW8, PLA2G2D, ANKRD2, RP11-529I10.4, HSPA12A, SGMS1, ASPM, SEC31B, NUDT13, MMACHC, CHMP2B, UPF2, ATRNL1, HERC4, LRIT1, EPC2, TCTN3, KIAA1279, PRDX5, FBXL2, TXN2, FAM26C, ANKK1, PCSK9, TCERG1L, ABL1, FTL, ALPL, FUT6, MSRB3, EDIL3, CDH11, ADAM8, PPIF, OPTN, CHST4, LHFP, CDKN1A, ZMPSTE24, CDKN1B, NET1, ABI1, LOC100129500, FRAT1, LOC100287804, CST8, SMNDC1, OLIG2, CBARA1, SEMA3A, ATP8A1, NDRG1, PEMT, ATP5H, VTI1B, ADARB2, CEBPA, SEMA4D, CEBPD, NOP56, NEBL, C10orf116, CCNI, SLC27A4, SLC27A2, LILRB4, LILRA1, LILRA2, C10orf10, AKR1C4, ATE1, BTN3A2, ZWINT, CAPN10, LDB3, SEC23IP, WIF1, AKAP2, PADI2, CHRM1, LEPREL2, UBD, GLRX3, PRDX4, AGPAT1, RPP30, RPP38, NPC2, TACC2, SORBS1, TXNRD2, POSTN, CELF2, CUGBP2, CDKN2B, RTN3, YME1L1, ARPP21, CPLX2, FRS3, FTCD, HSPA12B, DEFB118, ZMYND17, TTC18, BTBD16, ZNF488, PIK3AP1, ZFYVE27, COMTD1, C10orf4, ANKRD22, SFXN2, PDZD8, CSMD2, TUBGCP5, C10orf78, CLRN3, SFXN4, CPXM2, CD300A, PARK7, ECD, C12orf57, CHRNA3, BRD8, TSPAN9, TXNRD3, OSBPL7, OSBPL9, GRIN3A, GRIN3B, CLNK, CNTFR, COL1A1, COL6A1, COL6A2, TAF8, COL11A2, COL13A1, COL17A1, CPNE4, COMP, NIPA1, ADH1A, LTB4R, ADSSL1, FRMD6, CCR1, CNP, ADD3, CREBBP, CR2, KLF6, ADK, MAP3K8, PPARGC1B, COX10, COX15, CP, CPE, CPN1, CPT1A, CPT1B, DMBT1, ACAN, DIO1, DIO2, DLD, NKX2-3, DAB2, AKR1C1, DDIT3, MMAA, GLIS3, CLYBL, CYP2E1, CYP3A5, CYP11B2, USP54, CYP1A1, C6orf129, CYP2C19, CYP2C8, CTSZ, MAPK14, DYDC1, HECTD2, CSF1, CSF2, SLC2A14, CSK, A2ML1, CRHR1, MACROD2, ADAMTS14, C20orf61, CTNND2, CSPG4, TOM1L2, ADRA2B, CTSG, CTSH, CTSK, BTLA, C10orf129, SAMD8, TRUB1, MPP7, FRMPD2, ADRA1A, CTBP2, CTNNA2, RP5-1022P6.6, CTNNB1, EGR3, C10orf72, AHSG, EIF4EBP1, EIF4EBP2, AIF1, AGTR2, DVL1, EDN1, EDNRA, EDNRB, EFNA3, EFNA5, CELSR2, EGR2, DNTT, DOCK1, DOM3Z, DPP4, DPYSL2, DR1, DNM2, TRDMT1, DNMT3A, DUSP5, DRD5, JAG1, DRP2, RCAN1, RTKN2, C10orf27, MS4A2, ZNF438, SLC16A9, FAM13C, MARCH8, C10orf25, ZNF485, CCDC7, REEP3, JMJD1C, SLC39A12, C10orf107, TMEM26, ZCCHC24, ARMC3, UNC5B, C10orf35, TYSND1, ZNF25, F7, F10, F13A1, ABCD1, F3, ERCC2, EPHA4, ELN, MARK2, ENO2, SNCG, SLIT1, GRAMD3, SFTPA1, SLC6A12, SLC7A4, SLC8A1, BMP7, SLC11A1, SLC17A1, BMPR1A, SSX1, STAR, ND6, CYTB, ND5, ND4L, ND4, SOS2, SOX3, SP1, SP4, SPARC, SPP1, SRD5A2, BGLAP, C10orf84, SEL1L, MMS19, DNAJC1, TFB2M, CDH23, LHPP, PBLD, CLSTN2, TNMD, NPFFR1, SEPP1, MAP2K4, ABCG5, NOC3L, SFTPD, CSMD1, GAPDHL11, SHB, GMNC, P2RY12, BMP1, BMP3, SLC2A1, SLC2A4, BMP4, TIMP2, TLL2, THRB, TNFAIP1, TNFAIP2, TNFAIP3, TNFRSF1A, TFF2, THBD, TSPO, THBS4, TNNI3, TNXB, C1S, TRAF3, TRAF5, TRAF6, C4B, ATXN8, TTPA, NR2C2, TRAF1, TPT1, ABCC8, SVIL, VAMP2, STK11, STX1A, ATP8, trnK, COX2, COX1, SOD3, SOD1, TBP, TBXA2R, TBXAS1, HNF1A, ADAM17, TAF1, KLF5, ZEB1, MLX, SRF, TCN2, TECTB, PRDX2, TMBIM6, TM9SF3, PSMB9, ENTPD7, CAMK1D, ZMIZ1, ZNF248, PTBP1, PARD3, PSAP, SLC2A9, PSD, ASAH2, APOM, MAPK1, MAPK3, SEPT3, MAPK8, MAPK11, MAPK9, MAPK10, MAP2K1, MAP2K2, TDRD1, RUFY2, PRKCQ, TMEM106B, LRRC20, PPP2R4, WDR41, PPP2R5C, CWF19L1, PPP3CA, OLAH, SLC29A3, C10orf59, CRTAC1, ARMC4, PPP1R10, MTPAP, PAPD1, PPP2CA, CEP55, SEC61A2, PPP2R2A, BRWD2, FAM178A, CCAR1, OGDHL, CDK5RAP2, FAM63A, PRKCE, PAG1, SELS, CENPJ, CISD1, PI4K2A, MCM10, CSGALNACT2, H2AFY2, PRF1, DHTKD1, PRKAA1, ZNF673, PRKAB1, PRKAB2, HIF1AN, CCL4, CCL5, CCL8, CX3CL1, ATXN8OS, KLHL1AS, SERPINB4, BCL2, S100A6, S100B, REST, RET, BCL2L1, RFC1, RGR, RGS10, BCL3, AVPI1, RNASEL, EXOC4, BRD2, HPSE2, RNR1, ELAC2, RPS6KB1, RPS6KB2, RSU1, RXRB, RXRG, RYR3, BAG1, PTGER1, PTGER2, PTGER3, PTGER4, PTGFR, PTGIR, PTGIS, PTH, AS3MT, PTGS1, PTH1R, KIAA1161, ARID1B, ZNF608, GPR158, HECW2, SORCS2, STAMBPL1, ARHGAP21, KIAA1462, PTMS, PHF12, PYGB, PCTP, ARHGAP22, PZP, RAGE, ACTA2, RARA, RARB, RARG, KIAA1598, PTPN1, WDFY4, SEMA4G, TRIB3, PTPN6, ABCD4, PTPRE, EXOC6, UGT1A9, TREM1, POLG, KIAA1128, XRN1, POU2F1, SLC35F2, C10orf92, DCHS2, CNNM2, C10orf26, KIAA1797, PPP1R3A, PPP1R3C, PRKAG3, MYO3A, CNTN5, PNLIP, PNLIPRP1, PNLIPRP2, PNMT, TLR9, SERPINF2, LRP1B, PANK1, PI4KB, DUSP13, PCSK1, PCSK2, PCNT, HSPA14, ANKMY1, CD320, PIP4K2A, PITX3, PLA2G2A, PHEX, PHF1, PHYH, SERPINA1, PIK3CA, GALNAC4S-6ST, ANGPT4, PPME1, BIN2, LCMT1, REV1, PDE6C, PDE7A, CHMP5, PDGFRA, PDGFRB, ENPP1, SUFU, ATP5G2, ACSL5, GHRL, ATP8A2, CFP, PFKFB3, PFKP, NP, NOTCH3, NT3, NPPB, NPY2R, OTC, P2RX7, P4HA1, PAFAH1B1, PLA2G3, PRDX1, NRAP, NRF1, DDR2, C1orf66, ADIPOR1, TFB1M, MRPS16, GAL, PAX2, PARD6A, NTM, ATP5C1, PCBD1, CALY, REG3A, TNFRSF11B, OPRD1, TRNC, MTL1, TRNA, TRNF, ND4, ND4L, ND5, ND6, MS, NOTCH1, TRNH, NOS2, NINJ2, NODAL, NFATC4, NFE2L2, NFIB, NFKB1, NFKB2, NFRKB, TRNL1, TRNL2, TRNM, TRNN, TRNP, TRNQ, TRNR, TRNS1, TRNS2, TRNV, TRNW, TRNY, MUT, MX1, MXI1, MYC, SERPINC1, MYO10, NCAM2, NDUFA3, NDUFA6, NDUFA7, NDUFA8, NDUFA9, NDUFA10, NDUFAB1, NDUFB5, NDUFB7, NDUFB8, NDUFB9, NDUFB10, NDUFC2, NDUFS1, NDUFS2, NDUFS3, NDUFV1, NDUFS4, NDUFS5, NDUFS6, NDUFS8, NDUFV2, NDUFV3, NEDD4, MECP2, MEFV, MGC1, MGMT, CIITA, MICA, MICB, MKI67, NR3C2, MARK1, MARK3, MAT1A, COX3, CYTB, MRC1, MSR1, MSRA, MMP2, MMP16, MMP19, ING1, IL11, IL12A, ACADSB, IL15RA, IL17A, ITGA1, ITGA2, ITGA2B, ITGB1, ITIH2, JUNB, GSTK1, NDUFS7, NHLRC2, ARF1, KCNJ11, KLK1, KRT8, ACF, SLC16A12, CC2D2B, KDR, KIF5B, INSIG1, ITGA6, IREB2, LRP6, LRP5, ARL3, LTC4S, LTF, MIR124-1, M6PR, SMAD3, ARSA, ABLIM1, LHB, METTL10, FLJ44653, LOXL1, LPA, BLOC1S3, C12orf75, LCN1, LCT, L1CAM, ACAT2

Table 4. Complete list of 1551 genes used as the input gene set. Genes are ordered by the number of publications associating them with Alzheimer's disease listed in Phenopedia. First gene on the list has been mentioned in 1449 publication, the last 1123 genes have been mentioned in a single publication only.
